# The COngenital HeARt Disease in adult and Pulmonary Hypertension (COHARD-PH) registry: a descriptive study from single-center hospital registry of adult congenital heart disease and pulmonary hypertension in Indonesia

**DOI:** 10.1186/s12872-020-01434-z

**Published:** 2020-04-07

**Authors:** Lucia Kris Dinarti, Anggoro Budi Hartopo, Arditya Damar Kusuma, Muhammad Gahan Satwiko, Muhammad Reyhan Hadwiono, Aditya Doni Pradana, Dyah Wulan Anggrahini

**Affiliations:** Department of Cardiology and Vascular Medicine, Faculty of Medicine, Public Health and Nursing, Universitas Gadjah Mada – Dr. Sardjito Hospital, Yogyakarta, Indonesia

**Keywords:** Registry, Adult congenital heart disease, Atrial septal defects, Pulmonary hypertension

## Abstract

**Backgrounds:**

The COngenital HeARt Disease in adult and Pulmonary Hypertension (COHARD-PH) registry is the first registry for congenital heart disease (CHD) and CHD-related pulmonary hypertension (PH) in adults in Indonesia. The study aims to describe the demographics, clinical presentation, and hemodynamics data of adult CHD and CHD-related PH in Indonesia.

**Methods:**

The COHARD-PH registry is a hospital-based, single-center, and prospective registry which includes adult patients with CHD and CHD-related PH. The patients were enrolled consecutively. For this study, we evaluated the registry patients from July 2012 until July 2019. The enrolled patients underwent clinical examination, electrocardiography, chest x-ray, 6-min walking test, laboratory measurement, and transthoracic and transesophageal echocardiography. Right heart catheterization was performed to measure hemodynamics and confirm the diagnosis of pulmonary artery hypertension (PAH).

**Results:**

We registered 1012 patients during the study. The majority were young, adult females. The majority of CHD was secundum ASD (73.4%). The main symptom was dyspnea on effort. The majority of patients (77.1%) had already developed signs of PH assessed by echocardiography. The Eisenmenger syndrome was encountered in 18.7% of the patients. Based on the right heart catheterization, 66.9% of patients had developed PAH. Patients with PAH were significantly older, had lower peripheral oxygen saturation, had lower 6-min walking distance, and higher NTproBNP. The NTproBNP level independently predicted the development of PAH among CHD.

**Conclusions:**

The COHARD-PH registry is the first Indonesian adult-CHD and CHD-related PH registry. The demographics, clinical presentation, and hemodynamics dataof this registry reflect the situation in developing countries which needs to be compared with similar registries from developed countries.

## Background

The prevalence of adult congenital heart disease (CHD) in developed countries continues to rise due to improved survival attributed to successful surgical and medical management in childhood [1]. As a result, an increasing population of children with CHD is surviving into adulthood [2]. In less developed countries, a significant number of adults with CHD seeking medical help because of the emerging symptoms and signs of complications.

One of the devastating complications of CHD is pulmonary hypertension (PH) which occurs in about 10% of the CHD populations [3]. PH is defined as an increase in mean pulmonary artery pressures (mPAP) ≥25 mmHg at rest [4]. Based on the current clinical classification of PH, CHD may cause pulmonary artery hypertension (PAH) which is defined as a group 1 in this classification [4]. The PAH or PH group 1 is a clinical group which is characterised by hemodynamic parameters as pre-capillary PH (mPAP ≥25 mmHg with pulmonary artery wedge pressure (mPAWP) ≤15 mmHg) and pulmonary vascular resistance (PVR) > 3 Wood units (WU) [4]. The hemodynamic measurement by right heart catheterization (RHC) is mandatory to diagnose PAH and to assess the recommendation fordefect closure. The implications of CHD-related PH are limited functional capacity, increased risk of arrhythmias, right heart failure, and increased mortality [5].

The populational based registries in developed countries indicate that the prevalence of CHD-related PH is approximately 5–10% [6–8]. The CHD-related PH is a result of the systemic-to-pulmonary shunt at both the pre-tricuspid (atrial septal defect (ASD)) and the post-tricuspid levels (such as ventricle septal defect (VSD), patent ductus arteriosus (PDA), and aortopulmonary (AP) window) which cause the chronic increased flow to the pulmonary vessels. Its consequences are endothelial dysfunction, pulmonary vascular remodeling, increased pulmonary artery pressure and increased pulmonary vascular resistance [9].

Indonesia, a developing country and one of the most populous countries in the world, until recently did not have a national registry in regards to the CHD-related PH in adults. The prevalence and incidence of CHD-related PH are still unknown; nevertheless in clinical practice, adult patients with undetected and delayed diagnosis of CHD are frequent [10]. Compared with registries from developed countries, the situation regarding the adult CHD-related PH in developing countries are very different [11–13]. The COngenital HeARt Disease in adult and Pulmonary Hypertension (COHARD-PH) registry was initiated in 2012 to be the first registry done in Indonesia to describe adult CHD and CHD-related PH populations. This hospital-based registry is performed in Dr. Sardjito Hospital, Jogjakarta, Indonesia, which is a national referral hospital for cardiovascular disease in the region. The current study aims to describe the prevalence, demographics, clinical presentation, and hemodynamics characteristics of adult patients with CHD and CHD-related PH registered in the COHARD-PH registry.

## Methods

### Subjects

The COngenital HeARt Disease in adult and Pulmonary Hypertension (COHARD-PH) registry, is a single-center, observational, and prospective registry which enrolls adult patients with CHD and CHD-related PH. The adult patients presented in Dr. Sardjito Hospital, Jogjakarta, Indonesia with suspected CHD and CHD-related PH undergo a series of examinations to confirm the CHD and CHD-related PH diagnosis. The subjects are enrolled consecutively from outpatient clinics and inpatient wards. The enrollment and follow-up have been performed from July 2012 until the present. This study evaluated the patients of COHARD-PH registry enrolled from July 2012 until July 2019. This registry enrolled adult patients with age ≥ 18 years old.

### Procedures

Patients were interviewed, underwent physical examination, electrocardiography (ECG) examination, and chest x-ray examination. The suspected CHD patients continued for transthoracic echocardiography (TTE) as the initial examination to confirm the diagnosis of CHD. By TTE, the probability of PH was assessed based on current guidelines [4]. The bubble test was performed in selected cases if the TTE examination was dubious regarding septal defects/shunts. Transoesofageal echocardiography (TOE) was performed in patients with confirmed ASD and VSD by TTE examination. The TTE and TOE examination were done with G.E Vivid 7 (G.E Healthcare, U.S.A), G.E Vivid S6 (G.E Healthcare, U.S.A) or Phillips HD 15 (Philips N.V, The Netherland). The image acquisitions were made by three experience sonographers. The validation and confirmation of TTE and TOE examinations were performed by cardiologist consultants in our center dedicated to the registry. The cardiologist consultants had been tested for interobserver variability coefficients with results > 80% in agreement [14]. The image acquisition, validation and confirmation were in accordance with European Association of Echocardiography and American Society of Echocardiography guidelines prevailed in our hospital practice. The 6-min walking test to measure the distant of walking was performed for baseline of the registry.

Patients with simple defects such as ASD, VSD, PDA, AVSD, patent foramen ovale (PFO), and AP window were included in this study. The combined defects and other defect types were categorized as multiple defects. Patients with high probability of PH by TTE without confirmed CHD were excluded from the COHARD-PH registry (they were included in another PH registry). Complex CHD patients were excluded from the registry. The signs of Eisenmenger syndrome (desaturasion and bidirectional shunt from TTE) were noted and later confirmed by RHC.

**Fig. 1:**
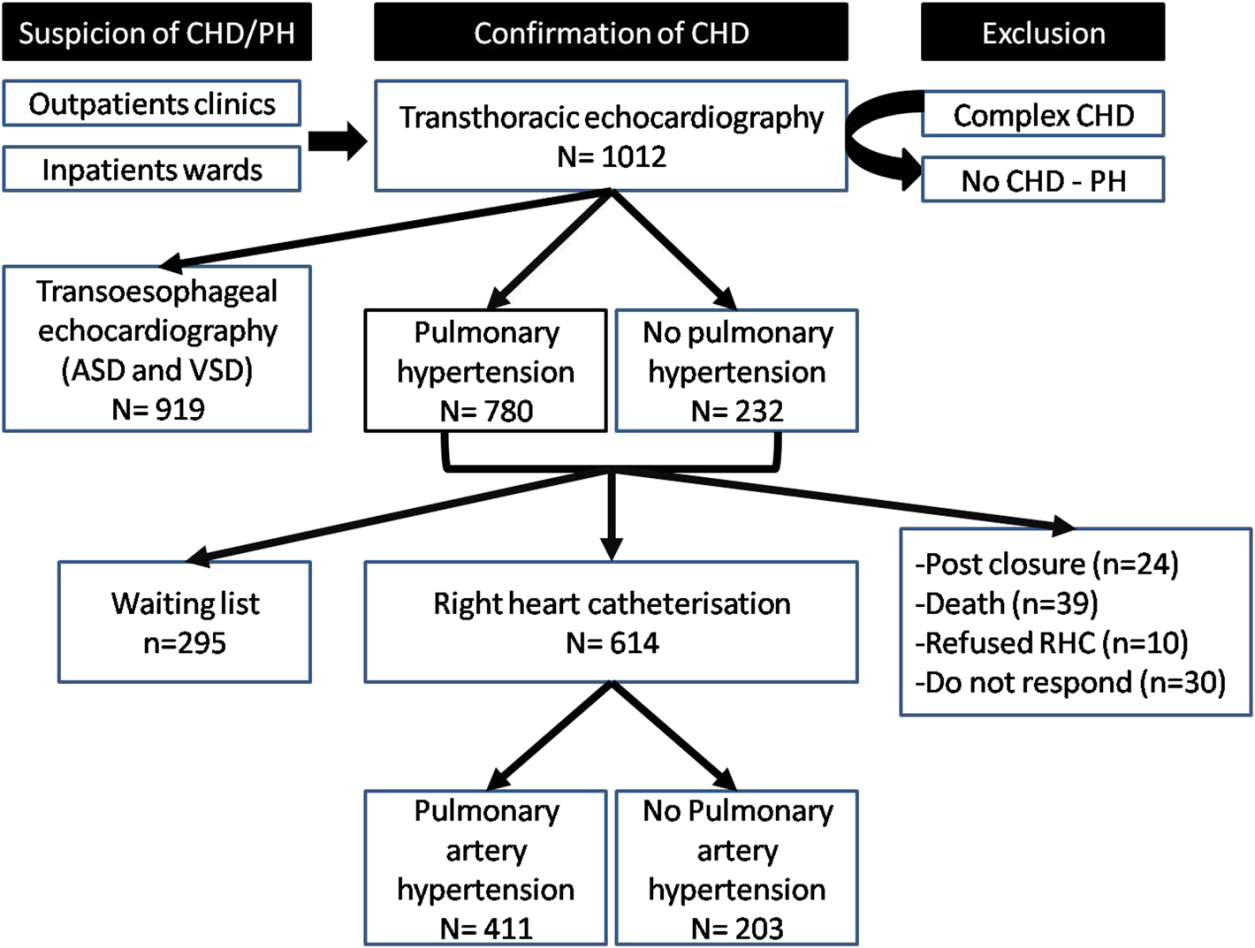
The flowchart of patients enrollment of COHARD-PH registry

Right heart catheterisation (RHC) was subsequently performed in all patients after being confirmed as CHD by TTE and TOE and enrolled for the registry. The RHC was performed by cardiologist consultants using standard procedures in non-sedated patients. The purpose of RHC was to measure hemodynamics, diagnose pulmonary artery hypertension (PAH) and decide the closure procedure for septal defects/shunts. The cardiac output was determined by indirect Fick method, as per hospital protocol. The flow ratio was calculated with the formula: pulmonary blood flow (Qp)/systemic blood flow (Qs) = (aorta saturation - mixed vein (MV) saturation)/(pulmonary vein (PV) saturation-pulmonary artery (PA) saturation). An MV saturation was calculated from: ((3 x superior vena cava saturation) + inferior vena cava saturation)/4. The pulmonary vascular resistance index (PVRi) was derived from the formula: (mPAP – mean left atrial pressure (mLAP) (or mPAWP)/Qp. A Qp was calculated from the formula: O_2_ consumption (ml/min)/(1.36x10xhemoglobin level x ((PV saturation-PA saturation)/100). The PVR was calculated from PVRi/body surface area. The PAH diagnosis was established when mPAP ≥25 mmHg, PVR > 3 WU and PAWP or mLAP ≤15 mmHg [4]. The diagnosis of Eisenmenger syndrome is established hemodynamically when Qp/Qs < 1 and PVRi > 8 WU.m^2^ [4]. The vasoreactivity test was performed in selected patients (discretion by cardiologist consultants). The vasoreactivity result was assessed based on current guideline (reduced in PVR > 20% and final PVRi < 6 WU.m^2^). The correctability of shunt was defined as patients with suitable anatomy of defects (surgery and/or device), Qp:Qs > 2 and PVRi < 6 WU.m^2^. Figure 1 shows the flowchart of COHARD-PH registry enrollment from July 2012 until July 2019.

The blood sample was collected from each patient by venipuncture in peripheral veins and during RHC. The blood sample was centrifuged and stored in − 80° for further analysis. The hemoglobin and hematocrit levels were measured with routine hemocytometer. The NTproBNP measurement was performed using a electrochemiluminescence immunoassay (ElecsysProBNP II) and a Cobas e immunoassay analyzer (Roche Diagnostics, Germany).

### Data collection

The research assistants dedicated to the registry collected and compiled the data and subsequently input the data to the electronic case report form of the COHARD-PH registry database. The baseline characteristics of patients were collected, comprising demographic data and clinical data. The ECG and chest X-ray results were documented. The TTE and TOE data were collected, comprising the type of CHD, dimension of right atrium (RA) and right ventricle (RV), left ventricle ejection fraction, tricuspid valvular regurgitation gradient (TVRG), tricuspid annular plane systolic excursion (TAPSE) and estimated mPAP. The 6-min walking distance was collected. The laboratory data were also compiled. The RHC data were collected, comprising mPAP, PAWP, mRAP, PVRi, mLAP, flow ratio, and oxygen saturations.

The signed informed consents were acquired for each patient to be included in the registry. The Medical and Health Research Ethics Committee of the Faculty of Medicine, Public Health and Nursing Universitas Gadjah Mada and Dr. Sardjito Hospital had approved the registry protocol.

### Statistics analysis

We performed the descriptive analysis of the data. The continuous data were presented in mean and standard deviation (SD) or median and interquartile range (IQR) depending on normality data distribution after tested with the Shapiro Wilk or Kolmogorov Smirnov test. The categorical data were presented in percentage. The comparison between two groups was conducted with Student T test and Chi-square test according to the type of data. The comparison among groups was conducted with one-way ANOVA test. A multivariable analysis was performed with logistic regression test to assess the independent predictor(s) among covariables. A value of *p* < 0.05 was set as statistically significant.

## Results

**Fig. 2:**
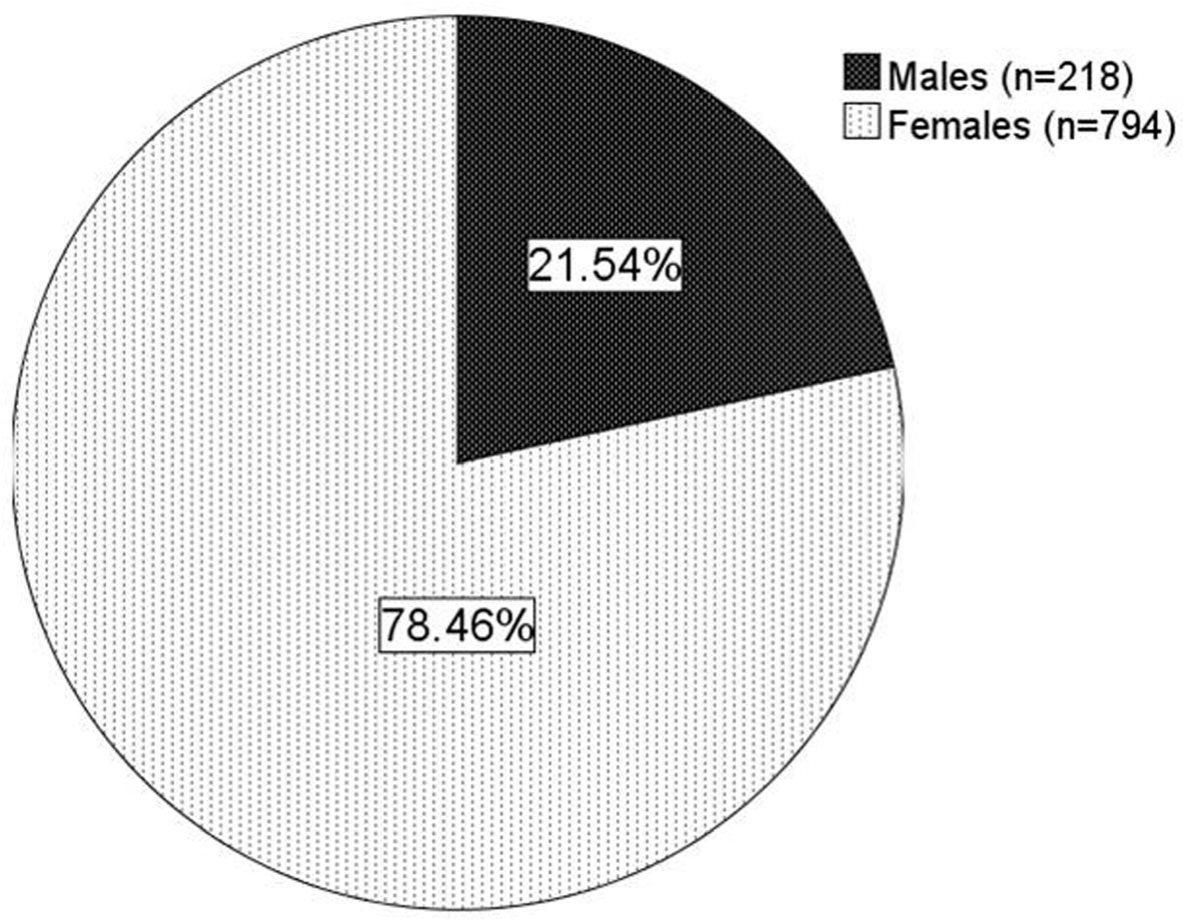
The proportion of sex in COHARD-PH registry. The majority were female patients (78.5%)

**Fig. 3:**
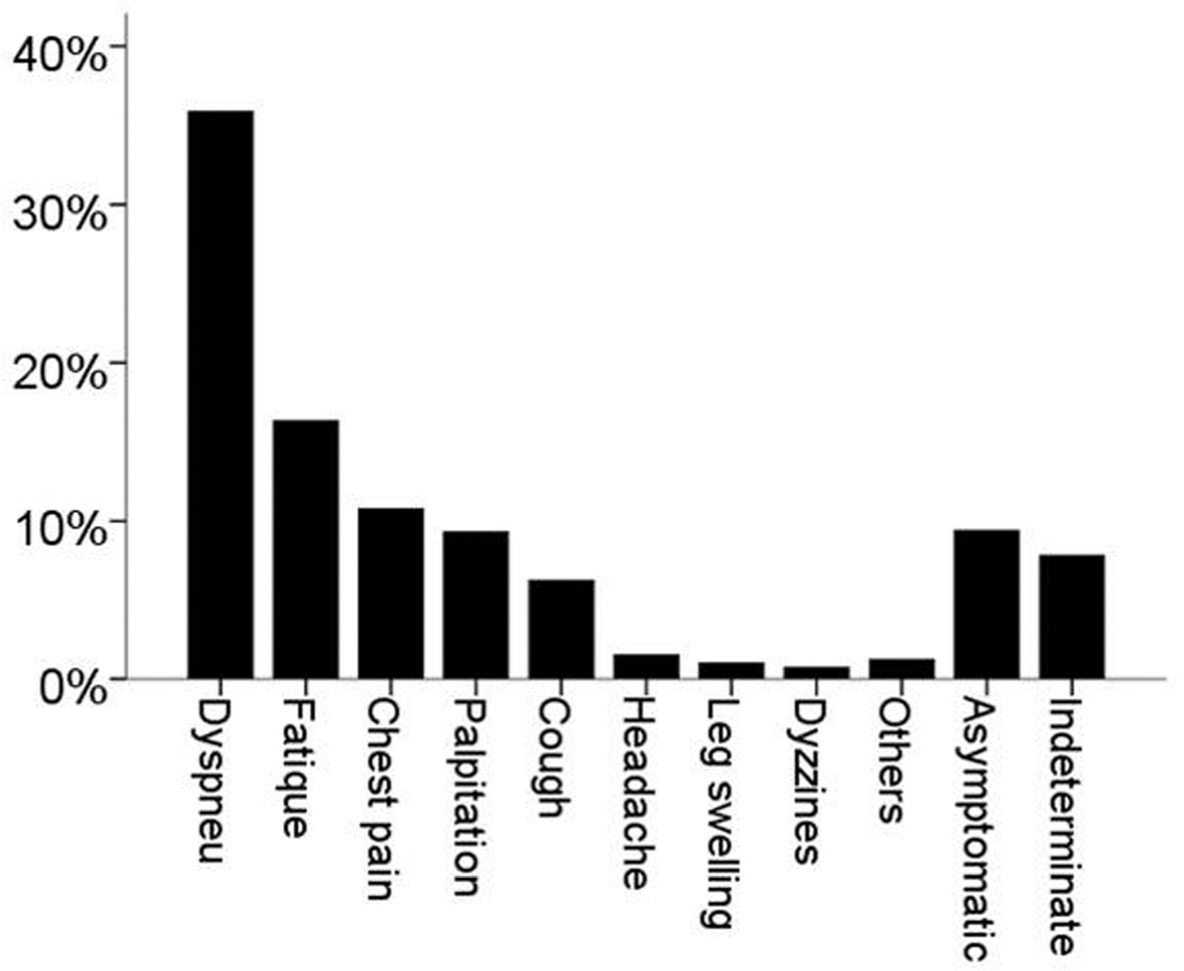
The percentage of symptoms of patients. Dyspnea on effort in 363 patients (35.9%), easily fatigued in 165 patients (16.3%), chest pain/discomfort in 109 patients (10.8%), palpitation in 94 patients (9.3%), cough in 63 patients (6.2%), headache in 15 patients (1.5%), leg swelling in 10 patients (1.0%), dizziness/syncope in 7 patients (0.7%), and other symptoms in 12 patients (1.2). Asymptomatic patients were 95 (9.4%) and indeterminate were 79 patients (7.8%)

From July 2012 until July 2019, we have registered data from 1012 patients who have the confirmed diagnosis as septal defects/shunts CHD. The clinical characteristics of the patients are shown in Table 1. The mean age of the patients at first diagnosis/enrollment was 34.7 years. The majority of patients were females, which accounted for 78.5% of all patients (as shown in Fig. 2). Normal and underweight body mass categories were predominant. Mean peripheral oxygen saturation was 95.5%. The WHO functional class was predominantly class II (43.0% of patients), only the minority of patients had worse WHO functional class (10.0% class III and 1.1% class IV). The mean 6-min walking distance was 356.5 m. The increased probability of PH by TTE examination was predominant (77.1%).The signs of Eisenmenger syndrome were encountered in 18.7% of patients. The laboratory results showed mean hemoglobin level was 13.8 g/dL, hematocrit 41.9% and median NTproBNP level 370.9 pg/mL. The main symptoms were dyspnea on effort (35.9%), easily fatigued (16.3%), chest pain/discomfort (10.8%) and palpitations (9.3%). As many as 9.4% of patients did not report any symptoms during first enrollment. The main symptoms of patients are depicted in Fig. 3.

**Table 1:** Demography, Clinical and Laboratory Characteristics of COHARD-PH Registry Patients

Characteristics	Total (***n*** = 1012)
Age at First Enrollment (years) [mean ± SD]	34.7 ± 13.2
Body Mass Index [mean ± SD]	19.9 ± 7.7
Underweight [n(%)]	417 (41.2)
Blood Pressure (mmHg) [mean ± SD]	
Systolic	111.9 ± 16.4
Diastolic	72.9 ± 11.4
Oxygen Saturation (%)[mean ± SD]^a^	95.5 ± 5.3
6 Minute Walk Distance (meter)[mean ± SD]^b^	356.5 ± 99.9
WHO Functional Class [n(%)]^c^	
I	418 (41.9)
II	435 (43.0)
III	101 (10.0)
IV	11 (1.1)
Hemoglobin (g/dL) [mean ± SD]^d^	13.8 ± 2.2
Hematocrit (%) [mean ± SD]^e^	42.1 ± 16.2
NTproBNP (pg/mL) [median (IQR)]^f^	370.9 (132.3–1625.0)
Pulmonary hypertension (by TTE) [n(%)]	780 (77.1)
Eisenmenger Syndrome [n (%)]	189 (18.7)
Post Defect Closure [n (%)]	24 (2.9)

^a^ data of 736 patients; ^b^ data of 616 patients; ^c^ data of 965 patients; ^d^ data of 574 patients; ^e^ data of 586; ^f^ data of 405 patients

The majority of CHD type was secundum ASD (73.4%). Other CHD types were perimembranous VSD (9.0%), PDA (5.8%), doubly-committed subarterial (DCSA) VSD (3.6%), sinus venosus ASD (2.0%), primum ASD (1.3%), PFO (0.8%), AVSD (0.3%) and AP window (0.1%). The patients with multiple defects accounted for 0.9% of all patients. The majority of patients had undergone RA and RV dilatation, with mean RA diameter of 45.6 mm and RV diameter of 42.1 mm. The mean mPAP based on TTE examination was 36.1 mmHg. The mean TVRG was 61.6 mmHg. The mean TAPSE was 24.3 mm. The mean left ventricle ejection fraction was 68.1%. Table 2 shows the results of TTE and TOE procedures.

**Table 2:** Echocardiography Characteristics of COHARD-PH Registry Patients

Echocardiographic Findings	Total (***n*** = 1012)
**Congenital anomaly** [n (%)]	
Primum ASD	13 (1.3)
Secundum ASD	743 (73.4)
Sinus venosus ASD	20 (2.0)
Multiple ASD	5 (0.5)
Patent foramen ovale	8 (0.8)
Perimembranous VSD	91 (9.0)
Doubly-committed subarterial VSD	36 (3.6)
Atrioventricular septal defect	3 (0.3)
Patent ductus arteriosus	59 (5.8)
Aortopulmonary window	1 (0.1)
Multiple defects	9 (0.9)
Postclosure ASD	18 (1.8)
Postclosure VSD	5 (0.5)
Postclosure PDA	1 (0.1)
**mPAP** (mmHg) [mean ± SD]^a^	35.3 ± 15.7
**TVRG** (mmHg) [mean ± SD]^b^	61.6 ± 34.3
**RA diameter** (mm) [mean ± SD]^c^	45.6 ± 8.8
**RV diameter** (mm) [mean ± SD]^d^	42.1 ± 9.0
**TAPSE (mm)** [mean ± SD]	24.3 ± 5.8
**Left ventricle EF** (%)[mean ± SD]	68.1 ± 8.8

^a^data from 828 subjects; ^b^ data from 810 subjects; ^c^ data from 843 subjects; ^d^ data 902 subjects

*ASD* atrial septal defect, *VSD* ventricular septal defect, *mPAP* mean pulmonary artery pressure, *TVRG* tricuspid valve regurgitation gradient, *RA* right atrium, *RV* right ventricle, *TAPSE* tricuspid annular plane systolic excursion, *EF* ejection fraction

The RHC had been performed in 614 subjects (60.7%). Among 1012 patients, 103 patients did not undergo RHC examination and 295 patients were on a waiting list to get RHC performed. The RHC was not performed in 103 patients due to: (1) patients had already had closure of defects (24 patients), (2) patients died before scheduled for RHC (39 patients), (3) patients refused the RHC examination (*n* = 10) and (4) patients did not respond to RHC schedule (30 patients). Patients who did not undergo RHC examination were mostly lost to follow-up from COHARD-PH registry and did not continue regular visits to our hospital. Patients who were on a waiting list were managed based on clinical symptoms and probability of PH based on echocardiography signs. The RHC results confirmed that 411 patients (66.9%) had developed PAH. The hemodynamics data from RHC showed median mPAP was 34.0 mmHg, PVRi 3.3 Wood Unit.m^2^, PAWP 10.0 mmHg, and flow ratio 2.3. The vasoreactivity test was performed in 186 patients and indicated that 43 patients (23.1%) had vasoreactive response. As many as 363 patients (59.1%) had correctable criteria for defect closure. Table 3 shows the result of RHC procedure.

**Table 3:** Hemodynamic Data from Right Heart Catheterizationof COHARD-PH Registry Patients

Right Heart Catheterization Result	Total (***n*** = 614)
**mPAP** (mmHg) [median (IQR)]	34.0 (23.0–56.0)
**PVRi** (Wood Unit.m^2^ )[median (IQR)]	3.3 (1.6–11.6)
**mRAP (mmHg)** [median (IQR)]^a^	9.0 (6.0–13.0)
**PAWP (mmHg)** [median (IQR)]^b^	10.0 (7.0–13.0)
**mLAP (mmHg)** [median (IQR)]^c^	10.0 (7.0–14.0)
**Flow Ratio** [median (IQR)]	2.3 (1.4–3.4)
**Aorta saturation (%)**[mean ± SD]^d^	91.9 ± 8.2
**SVC saturation (%)**[mean ± SD]^e^	62.9 ± 9.6
**IVC saturation (%)**[mean ± SD]^f^	72.8 ± 9.9
**Left atrial saturation (%)**[mean ± SD]^g^	91.7 ± 5.7
**Right atrial saturation (%)**[mean ± SD]^h^	77.6 ± 9.9
**Right ventricle saturation (%)**[mean ± SD]^h^	80.2 ± 11.6
**Pulmonary artery saturation (%)**[mean ± SD]^i^	81.4 ± 10.0
**Pulmonary vein saturation (%)**[mean ± SD]^j^	94.1 ± 5.9
**PAH** [n (%)]	411 (66.9)
**Vasoreactivity test** [n (%)]^k^	
-Vasoreactive	43 (23.1)
-Non vasoreactive	143 (76.9)
**Correctable** [n (%)]	363 (59.1)

^a^ data of 563 patients; ^b^ data of 219 patients; ^c^ data of 517 patients; ^d^ data of 467 patients; ^e^ data of 507 patients; ^f^ data of 457 patients; ^g^ data of 418 patients; ^h^ data of 456 patients; ^i^ data of 499 patients; ^j^ data of 410 patients; ^k^ performed in 186 patients

*mPAP* mean pulmonary artery pressure, *PVRi* pulmonary vascular resistance index, *mRAP* mean right atrial pressure, *PAWP* pulmonary artery wedge pressure, *mLAP* mean left atrial pressure, *SVC* superior vena cava, *IVC* inferior vena cava, *PAH* pulmonary arteryhypertension

Table 4 shows the comparison of clinical and laboratory parameters between patients with CHD-related PAH and those without PAH. Patients with PAH had significantly older age at first diagnosis (36.4 ± 12.9 vs. 32.2 ± 12.0 years old, *p* < 0.001), lower peripheral oxygen saturation (94.8 ± 5.5 vs. 97.4 ± 3.2%, p < 0.001), lower 6-min walking distance (336.3 ± 99.7 vs. 393.9 ± 82.1 m, *p* < 0.001), worse WHO functional class (WHO III-IV: 14.2% vs. 5.0%, *p* < 0.001), higher hemoglobin level (14.1 ± 2.2 vs. 13.5 ± 1.9 g/dL, *p* = 0.006), higher hematocrit level (42.2 ± 6.5 vs. 40.2 ± 4.9%, *p* < 0.001) and higher NTproBNP level (median: 774.0 vs. 121.5 pg/mL, *p* < 0.001). The proportion of ASD was predominant in patients with PAH (89.3%), followed by PDA (5.1%) and VSD (4.1%). Among the patients with multiple defects, the majority had developed PAH (4 of 5 patients) and all subjects with AP window and AVSD had PAH. Multivariable analysis showed that only NTproBNP level independently predicts the PAH in patients with CHD (OR 1.003, 95% CI: 1.001–1.004, *p* = 0.001), as shown in Table 5.

**Table 4:** Characteristics of Patients based on the PAH Diagnosis by RHC (*n* = 614)

Characteristics	No PAH (***n*** = 203)	PAH (***n*** = 411)	***P*** value
**Age at Enrollment** (years) [mean ± SD]	32.2 ± 12.0	36.4 ± 12.9	< 0.001
**Gender** [n (%)]			
Males	42 (20.7)	75 (18.2)	0.469
Females	161 (79.3)	336 (81.8)	
**Body Mass Index** [mean ± SD]	20.2 ± 3.4	19.5 ± 6.9	0.136
**Blood Pressure** (mmHg) [mean ± SD]			
Systolic	111.4 ± 15.1	110.7 ± 18.8	0.678
Diastolic	73.4 ± 10.9	72.9 ± 11.9	0.698
**Oxygen Saturation** (%)[mean ± SD]^a^	97.4 ± 3.2	94.8 ± 5.5	< 0.001
**6 Minute Walk Distance** (meter)[mean ± SD]^b^	393.9 ± 82.1	336.3 ± 99.7	< 0.001
**WHO Functional Class** [n(%)]^c^			< 0.001
I	122 (60.4)	136 (34.0)	
II	70 (34.7)	207 (51.8)	
III-IV	10 (5.0)	57 (14.2)	
**Hemoglobin** (g/dL) [mean ± SD]^d^	13.5 ± 1.9	14.1 ± 2.2	0.006
**Hematocrit** (%) [mean ± SD]^e^	40.2 ± 4.9	42.2 ± 6.5	< 0.001
**NTproBNP (pg/mL)** [median (IQR)]^f^	121.5 (57.1–218.1)	774.0 (242.8–2022.3)	< 0.001
**Congenital Anomalies** [n (%)]			0.005
ASD	166 (81.8)	367 (89.3)	
VSD	26 (12.8)	17 (4.1)	
PDA	10 (4.9)	21 (5.1)	
Multiple defect	1 (0.5)	4 (1.0)	
AP window	0 (0)	1 (0.2)	
AVSD	0 (0)	1 (0.2)	

^a^ data of 488 patients, ^b^ data of 442 patients, ^c^ data of 602 patients, ^d^ data of 439 patients, ^e^ data of 444 patients, ^f^ data of 294 patients,

*PAH* pulmonary artery hypertension, *ASD*: atrial septal defect, *VSD* ventricular septal defect, *PDA* patent ductus arteriosus, *AP* aortopulmonal, *AVSD* atrioventricular septal defects, *WHO* world health organisation

**Table 5:** Multivariable Analysis to Predict the PAH

Covariables*	OR	95%CI	***P*** value
**Age at Enrollment**	1.020	0.986–1.055	0.258
**Oxygen Saturation**	0.829	0.686–1.001	0.052
**6 Minute Walk Distance**	0.997	0.993–1.001	0.206
**WHO Functional Class (III-IV)**	1.874	0.322–10.886	0.484
**Hemoglobin** (g/dL)	1.019	0.581–1.789	0.947
**Hematocrit** (%)	0.952	0.781–1.160	0.627
**NTproBNP (pg/mL)**	1.003	1.001–1.004	0.001
**Congenital Anomalies (ASD)**	0.778	0.219–2.767	0.698

*Covariables included were those with *p* < 0.05 from Table 4

Table 6 shows the difference of characteristics among patients based on WHO functional class (total amount 602 patients). Worse WHO functional class (class III-IV) was marked by the least peripheral oxygen saturation, the least 6-min walk distance and the highest NTproBNP level. Based on echocardiography examination, worse WHO functional class was associated with increased mPAP, higher TVRG, larger RA and RV diameters, lower TAPSE and lower LVEF. Based on RHC results, worse WHO functional class was related with higher mPAP and increased PVRi. The ASD patients were the majority among those with worse WHO functional class (95.5%).

**Table 6:** Characteristics of Patients based on the WHO Functional Class (*n* = 602)

Characteristics	WHO class I (***n*** = 258)	WHO class II (***n*** = 277)	WHO class III-IV (***n*** = 67)	***P*** value
**Age at Enrollment **(years) [mean ± SD]	34.5 ± 12.9	34.9 ± 12.8	36.6 ± 12.2	0.498
**Gender** [n (%)]				
Males	55 (21.3)	53 (19.1)	7 (10.4)	0.131
Females	203 (78.7)	224 (80.9)	60 (89.6)	
**Body Mass Index** [mean ± SD]	19.9 ± 3.4	19.2 ± 3.9	20.9 ± 14.7	0.064
**Oxygen Saturation** (%)[mean ± SD]^a^	96.9 ± 3.2	95.4 ± 5.2	92.6 ± 8.1	< 0.001
**6 Minute Walk Distance** (meter)[mean ± SD]^b^	403.9 ± 80.2	333.1 ± 88.4	276.6 ± 104.6	< 0.001
**Hemoglobin** (g/dL) [mean ± SD]^c^	13.8 ± 2.0	13.9 ± 2.1	14.0 ± 2.5	0.569
**Hematocrit** (%) [mean ± SD]^d^	41.4 ± 5.3	41.6 ± 6.4	41.9 ± 7.6	0.783
**NTproBNP (pg/mL)** [median (IQR)]^e^	178.0 (91.9–718.6)	556.9 (159.6–1687.0)	929.1 (383.0–2882.0)	0.022
**Congenital Anomaly** [n (%)]				0.001
ASD (*n* = 523)	207 (80.2)	252 (91.0)	64 (95.5)	
VSD (*n* = 41)	29 (11.2)	9 (3.2)	3 (4.5)	
PDA (*n* = 31)	21 (8.1)	10 (3.6)	0 (0)	
Others (*n* = 7)	1 (0.5)	6 (2.2)	0 (0)	
**Echocardiography parameters**				
** mPAP** (mmHg) [mean ± SD]^f^	32.6 ± 14.7	37.3 ± 15.2	43.5 ± 15.0	< 0.001
** TVRG** (mmHg) [mean ± SD]^g^	50.7 ± 32.7	66.5 ± 34.1	80.8 ± 35.4	< 0.001
** RA diameter** (mm) [mean ± SD]^h^	44.8 ± 7.5	46.6 ± 8.1	47.4 ± 8.6	0.017
** RV diameter** (mm) [mean ± SD]^i^	39.8 ± 7.7	44.4 ± 8.4	45.4 ± 7.5	< 0.001
** TAPSE (mm)** [mean ± SD]^j^	25.4 ± 5.3	25.2 ± 5.8	22.7 ± 5.4	0.002
** Left ventricle EF** (%)[mean ± SD]^k^	68.9 ± 7.4	69.7 ± 9.1	66.1 ± 11.6	0.011
**RHC parameters**				
**mPAP** (mmHg) [median (IQR)]^l^	28.0 (20.3–46.0)	40.0 (26.0–58.0)	52.5 (32.8–68.5)	< 0.001
**PVRi** (Wood Unit.m^2^) [median (IQR)]^m^	2.2 (1.4–5.3)	3.8 (1.5–13.5)	10.2 (2.1–21.6)	< 0.001
**Flow Ratio** [median (IQR)]^n^	2.5 (1.7–3.6)	2.4 (1.5–3.4)	1.8 (1.0–3.1)	0.115

^a^ data of 488 patients;^b^ data of 442 patients; ^c^ data of 439 patients;^d^ data of 444 patients;^e^ data of 294 patients;^f^ data of 504 patients;^g^ data of 519 patients; ^h^ data of 542 patients; ^i^ data of 566 patients; ^j^ data of 572 patients; ^k^ data of 581 patients; ^l^ data of 582 patients; ^m^ data of 515 patients;^n^ data of 530 patients;

*WHO* world health organisation, *PAH* pulmonary artery hypertension, *ASD* atrial septal defect, *VSD* ventricular septal defect, *PDA* patent ductus arteriosus, *AP* aortopulmonal, *AVSD* atrioventricular septal defects, *mPAP* mean pulmonary artery pressure, *TVRG* tricuspid valve regurgitation gradient, *RA* right atrium, *RV* right ventricle, *TAPSE* tricuspid annular plane systolic excursion, *EF* ejection fraction, *mPAP* mean pulmonary artery pressure, *PVRi* pulmonary vascular resistance index

**Fig. 4:**
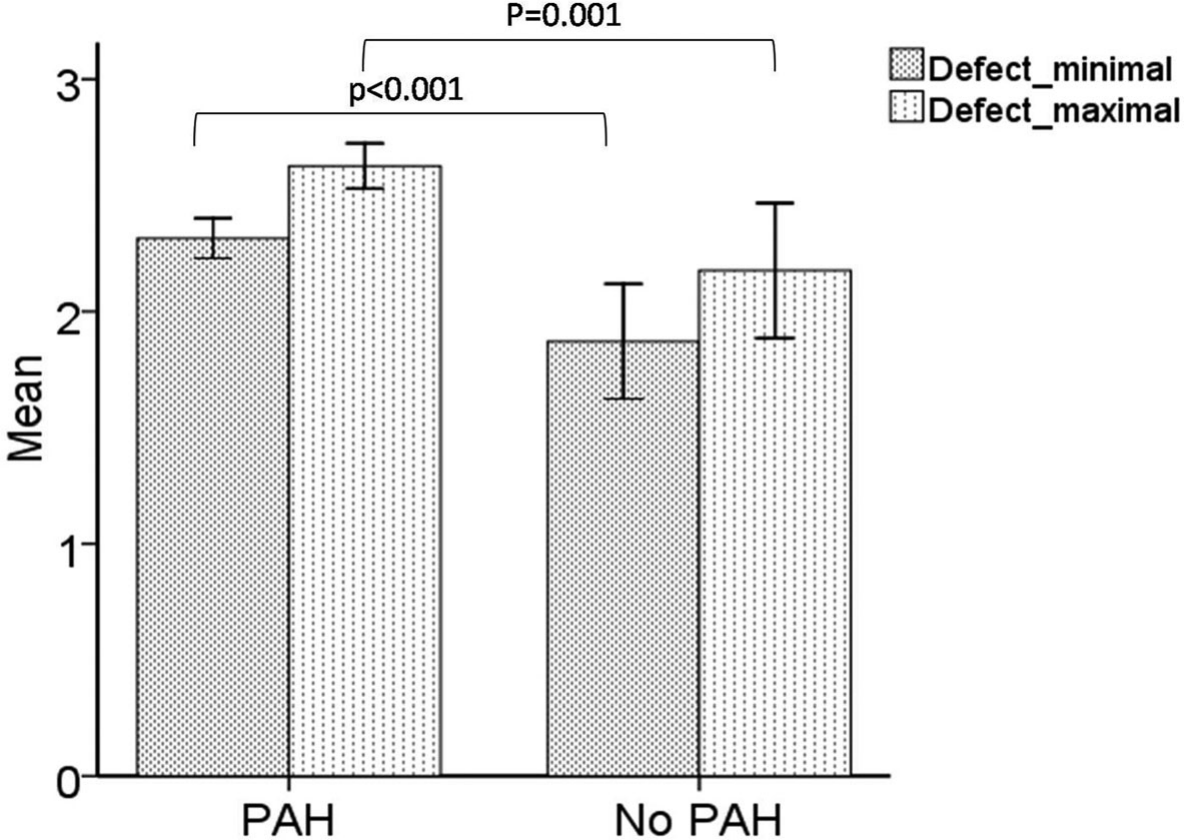
The PAH development associated with defect diameters in ASD. Minimum defect (2.3 ± 0.8 vs. 1.9 ± 1.5 cm, *p* < 0.001) and maximum defect (2.6 ± 0.9 vs. 2.2 ± 1.8 cm, *p* = 0.001) between ASD patients with PAH (*n* = 341) and those without PAH (*n* = 157)

**Fig. 5:**
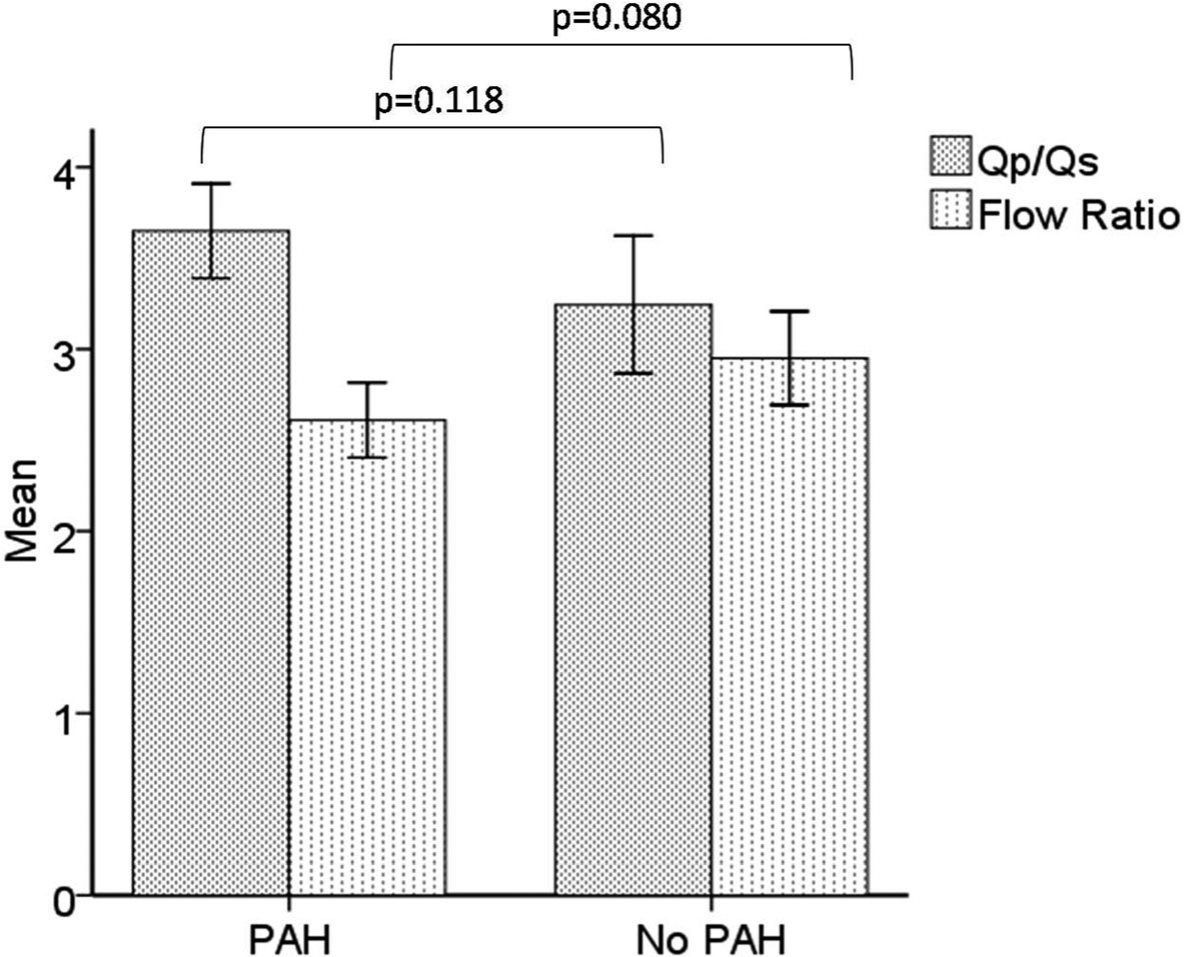
The PAH development did not associate with shunt flow in ASD. The Qp:Qs ratio by TTE (3.6 ± 2.2 vs. 3.3 ± 2.2, *p* = 0.118) between ASD patients with PAH (*n* = 306) and those without PAH (*n* = 138) and flow ratio by RHC (2.6 ± 1. 3vs. 2.8 ± 1.4, *p* = 0.080), between ASD patients with PAH (*n* = 326) and those without PAH (*n* = 137)

The predominance of ASD patients in the COHARD-PH registry was in accord with previous reports. The development of PAH in ASD patients may be associated with defect size and shunt flow. We analysed the difference of minimal and maximal diameter of ASD defect based on echocardiography examination between ASD patients with PAH and those with no PAH. The ASD patients with PAH had larger minimal defect diameter as compared to those without PAH (2.3 ± 0.8 vs. 1.9 ± 1.5 cm, *p* < 0.001) and larger maximal defect diameter (2.6 ± 0.9 vs. 2.2 ± 1.8 cm, *p* = 0.001) (as shown in Fig. 4). There was no significant difference in the Qp/Qs ratio based on TTE and flow ratio based on RHC results between ASD patients with PAH and those without PAH (as shown in Fig. 5).

There was an incremental increase of the proportion of PAH according to age range, with the highest proportion of PAH in the age group between 51 and 60 years old (Table 7).

**Table 7:** The Age Distribution Based of CHD-associated PAH

Age (years)	Congenital heart diseases (n (%))	PAH (n (%))
17–20	72 (11.7)	41 (56.9)
21–30	194 (31.6)	121 (62.4)
31–40	156 (25.4)	101 (64.7)
41–50	108 (17.6)	80 (74.1)
51–60	61 (9.9)	51 (83.6)
> 61	23 (3.7)	17 (73.9)
**Total**	614	411

*CHD* congenital heart disease, *PAH* pulmonary artery hypertension

## Discussion

We report the first hospital-based registry of adults with CHD and CHD-related PH in Indonesia which is comprised of the complete diagnostic work-up. The COHARD-PH registry is a single-center registry in Dr. Sardjito Hospital, a national referral center for cardiovascular disease in Indonesia. The COHARD-PH registry was started in July 2012 and continues until the present day. Within the duration of 7 years, July 2012 – July 2019, the number of patients enrolled were 1012 adults with CHD. The majority of CHD in this registry are ASD, followed by VSD and PDA. Young adult females (ages between 21 and 40 years old) are predominant in our registry. Most patients are symptomatic, with the majority in WHO functional class II. By echocardiography, the prevalence of increased probability of PH is 77.1%.With the further confirmation by RHC measurement, the COHARD-PH registry shows that 66.9% subjects had developed PAH.

In our registry, the majority of CHD is ASD. This is similar with another study in an adult registry, that showed ASD is the most common [1]. Most patients come to our medical facility when they already had a complaint, with shortness of breath and easily fatigued among the most common complaints. These symptoms are similar to previous studies where the patients are already in an advanced condition and had limited activity [13, 15]. Since the most prevalent CHD in our registry is ASD, the patients remained asymptomatic for decades. Therefore, the symptoms that appear later will urge the patients to consult doctor and visit hospital. The main symptoms of patients were associated with the development of PAH, which was later confirmed by RHC procedure. We excluded patients with complex adult CHDs from the analysis because most of them were already diagnosed in childhood and the proportion of PH and PAH cannot accurately be determined. Furthermore, complex adult CHDs have distinct characteristics in association with development of PH and PAH.

The early finding of CHD-related PAH is often not easy to recognize due to the unknown precise period of PAH [11]. The chronic systemic-to-pulmonary shunt is a congenital malformation causing blood overflow in the pulmonary vasculature from infancy, and if left untreated may give rise to PAH in adult life. The majority of our patients were untreated cases, and probably undetected cases in their childhood period. They presented to our hospital late in their delayed and progressed diseases.

Our registry shows that patients are predominantly young adult females. This observation is consistent with other registries [3, 6, 7, 13, 16–18]. The mean age of patients in our registry during first enrollment is 34.7 years old. The fact is that most cases are ASDs, in which at younger age there had been no complaints. In ASD clinical presentation, the pulmonary hypercirculation and right heart volume overload induce PAH after a longer period of time, which is different from VSD or PDA [6]. The patients with VSD and PDA are symptomatic in earlier years of age and have more evident signs, probably before reaching adulthood, therefore they are mostly detected in childhood and adolescent period [12]. Our hospital registry in pediatric patients indicated that VSD has the most prevalence in childhood [19]. Moreover, 72.7% of patients are asymptomatic for a long period of time (> 2 decades). Mostly in the third decades of life, the PAH complications start to clinically manifest and urge the patients to visit the hospital.

The echocardiographic data showed that the majority of the patients are categorized in increased probability of PH and confirmed PAH by RHC, which is a gold standard for diagnosis of PH and PAH. Almost 70% of our patients have already developed PAH based on RHC. These data are much higher than data from other registries, especially registries from developed countries. The striking difference is likely due to late presentation and selection bias, because the patients are enrolled at our hospital mostly due to signs and symptoms they suffer. Currently, in Indonesia there is no screening and early detection of asymptomatic CHD, therefore many patients are undetected until they come to visit medical facilities due to complications.

The patients with PAH have worse clinical characteristics as compared to those without PAH. They are in young adult age and older than patients without PAH. Their functional capacities, measured by WHO class, 6-min walking distance and peripheral oxygen saturation, are worse. The NTproBNP levels, the sole biomarker for prognostication of PH, are higher in patients with PAH as compared with those without. Among CHD-associated PAH, it should be noted that four different clinical subgroups have been proposed which reflect different pathophysiology and prognosis [4, 12, 17]. Patients with small defect-associated PAH had similar outcome with Eisenmenger syndrome which was better than the outcome of patients in which PAH develops or persists after closure of the defect [17, 18]. The large defects with prevalent systemic-to-pulmonary shunts have better survival as compared with other clinical types [12, 17].

### Limitation

The COHARD-PH registry is a hospital-based registry therefore patients who enrolled were those who developed symptoms. Although they cannot represent the entire population in the community, the registry reflects real world conditions of earlier undetected congenital heart disease in Indonesia. The procedure for hemodynamic evaluation, i.e. RHC, was limited by hospital standard procedures and timeframe, therefore not all patients in the registry underwent complete RHC. However, the fact that the majority of patients had undergone RHC is an accomplishment of this registry.

## Conclusions

The COHARD-PH registry is the first reported Indonesian adult CHD and CHD-related PH registry. The demographics data, clinical presentation, and hemodynamics data of this hospital-based registry are indicative of the real world situation in developing countries which needs to be compared with other CHD-related PH and PH registries, both from developed and other developing countries.

## Data Availability

The datasets used and/or analysed during the current study are de-identified and available from the corresponding author on reasonable request.

## Abbreviations

AP: Aortopulmonary
ASD: Atrial septal defect
CHD: Congenital heart disease
COHARD-PH: The COngenital HeARt Disease in adult and Pulmonary Hypertension
DCSA: Doubly-committed subarterial
ECG: Electrocardiography
IQR: Interquartile range
mLAP: Mean left atrial pressure
mPAP: Mean pulmonary artery pressures
mRAP: Mean right atrial pressure
PDA: Patent ductus arteriosus
PFO: Patent foramen ovale
PH: Pulmonary hypertension
PVR: Pulmonary vascular resistance
PVRi: Pulmonary vascular resistance index
RA: Right atrial
RHC: Right heart catheterization
RV: Right ventricle
Qp: Pulmonary blood flow
Qs: Systemic blood flow
SD: Standard deviation
TOE: Transoesofageal echocardiography
TTE: Transthoracal echocardiography
TVRG: Tricuspid valvular regurgitation gradient
VSD: Ventricle septal defect
WU: Wood units
